# Impact of Educational Level on Performance on Auditory Processing Tests

**DOI:** 10.3389/fnins.2016.00097

**Published:** 2016-03-10

**Authors:** Cristina F. B. Murphy, Camila M. Rabelo, Marcela L. Silagi, Letícia L. Mansur, Eliane Schochat

**Affiliations:** Department of Physical Therapy, Speech-Language Pathology and Occupational Therapy, School of Medicine, University of São PauloSão Paulo, Brazil

**Keywords:** education, auditory processing, cognition, elderly, behavioral tests, electrophysiological tests

## Abstract

Research has demonstrated that a higher level of education is associated with better performance on cognitive tests among middle-aged and elderly people. However, the effects of education on auditory processing skills have not yet been evaluated. Previous demonstrations of sensory-cognitive interactions in the aging process indicate the potential importance of this topic. Therefore, the primary purpose of this study was to investigate the performance of middle-aged and elderly people with different levels of formal education on auditory processing tests. A total of 177 adults with no evidence of cognitive, psychological or neurological conditions took part in the research. The participants completed a series of auditory assessments, including dichotic digit, frequency pattern and speech-in-noise tests. A working memory test was also performed to investigate the extent to which auditory processing and cognitive performance were associated. The results demonstrated positive but weak correlations between years of schooling and performance on all of the tests applied. The factor “years of schooling” was also one of the best predictors of frequency pattern and speech-in-noise test performance. Additionally, performance on the working memory, frequency pattern and dichotic digit tests was also correlated, suggesting that the influence of educational level on auditory processing performance might be associated with the cognitive demand of the auditory processing tests rather than auditory sensory aspects itself. Longitudinal research is required to investigate the causal relationship between educational level and auditory processing skills.

## Introduction

Research has revealed a positive impact of education on cognitive skills among adults and elderly people (Blum and Jarvik, 1974; Gurland et al., 1983; Evans et al., 1993; Farmer et al., 1995; Leibovici et al., 1996; Brucki et al., 2003; Souza-Talarico et al., 2007; Zahodne et al., 2011). For instance, some studies have demonstrated a strong correlation between levels of education and performance on tests of cognitive function (Blum and Jarvik, 1974; Gurland et al., 1983; Farmer et al., 1995; Brucki et al., 2003; Souza-Talarico et al., 2007). Others have investigated the extent to which educational levels might affect the course of cognitive decline associated with aging (Evans et al., 1993; Leibovici et al., 1996; Zahodne et al., 2011). Evans et al. (1993) demonstrated that, regardless of age, birthplace and occupation, elderly people with fewer years of formal education exhibit greater declines in cognitive function, leading to the hypothesis that low levels of education might be associated with the development of Alzheimer's disease and dementia in general.

Overall, the positive effect of education is related to the “cognitive reserve theory,” which suggests that environmental enrichment leads to an increase in the number of synapses and vascularization, which leads to changes in the structure of the brain early in life (Speisman et al., 2013). However, an alternative hypothesis is that the positive effect is explained by the likely correlation between education and health (Albert, 1995). According to Albert (1995), people with less education tend to have greater exposure to risks, such as occupational exposure and unhealthy habits, and these issues might affect not only cognitive function but also sensory skills over time.

Although the positive effect of education on cognitive skills is currently widely accepted, the impact of education on auditory processing has not yet been evaluated. This topic is reasonable given the studies demonstrating sensory-cognitive interactions in the aging process (Peters et al., 1988; Baltes and Lindenberger, 1997; Panza et al., 2015; Wayne and Johnsrude, 2015). One example of this sensory-cognitive interaction is the correlation between cognitive performance in working memory tests and some auditory processing skills, such as speech-in-noise perception (Pichora-Fuller et al., 1995; Akeroyd, 2008; Füllgrabe et al., 2015), pitch pattern frequency (Mukari et al., 2010) and dichotic listening tests (Hällgren et al., 2001). Regarding speech-in-noise perception, Pichora-Fuller (2003) hypothesized that the efficient operation of the working-memory system becomes compromised, negatively affecting the comprehension of spoken language as a consequence of hearing difficulties and the effort required to listen in the presence of noise. Dichotic listening performance has also been associated with working memory skills, especially in the forced-left condition that requires a great cognitive engagement produced by competition with “right ear advantage” (Hugdahl and Anderson, 1986; Hugdahl et al., 2001; Hugdahl, 2003). Studies have also reported an association between sensory declines, such as presbycusis and auditory processing disorders, and cognitive declines, such as mild cognitive impairment and dementia (Peters et al., 1988; Baltes and Lindenberger, 1997; Avila et al., 2014; Panza et al., 2015; Wayne and Johnsrude, 2015).

From a neurophysiological perspective, this sensory-cognitive interaction is based on the significant contribution of the top-down mechanisms of auditory perception, which is supported by the involvement of multi-modal association areas of the cortex in response to simple sounds and the contribution of the efferent auditory system in modulating some auditory processing skills, such as binaural processing (Moore, 2012). Thus, because both sensory and cognitive factors are strongly involved, we might predict that, as long as education leads to improved cognitive performance or affects the course of cognitive decline, it will also be possible to observe improved performance on tests involving auditory processing skills.

To investigate this issue, we aimed to determine the extent to which years of formal schooling were associated with the performance of people on auditory processing tasks. Middle-aged and elderly people were included based on the hypothesis that education might affect the course of cognitive decline associated with aging, and, consequently, the course of the decline in auditory processing as well. Obviously, it will not be possible for the present study to directly address this topic given that it was not a longitudinal study; however, as long as education correlates with performance on auditory processing tests, the present findings might be the basis for further follow-up studies in which the influence of education on auditory processing declines might be properly addressed. The present study also has important clinical implications given that, until the present time, the variable “educational level” was not taken into consideration at the time of auditory processing diagnoses.

The association between educational level and auditory processing performance was investigated using auditory processing (dichotic digit, speech-in-noise, and frequency pattern) measures. We also added a working memory task to investigate the extent to which better performance on the auditory processing tests was associated with enhanced cognitive skills. Accounting for the hypothesis that higher education exerts a positive impact on auditory processing skills, we predicted a significant contribution of education on the variance of the auditory processing tests performance. We expected the results to contribute to a better understanding of the benefits associated with education in healthy adults and elderly people.

## Methods

### Ethics statement

This study was conducted at the Department of Physical Therapy, Speech-Language Pathology and Occupational Therapy at the School of Medicine at the University of São Paulo and was approved by the Research Ethics Committee for the Analysis of Research Projects at the University Hospital Medical School, University of São Paulo, under protocol number CEP-HU/USP: 100511 0 -SISNEP CAAE: 0034.0.198.000-10. A written consent form with detailed information about the aim and protocols of the study was also approved by this ethics committee.

### Participants

A total of 177 adults, aged 50–87 years took part in the study. Education levels varied from 0 to 24 years. The effect of educational level was investigated using the “years of formal schooling”; thus, the more years studied, the higher the level of education achieved. The inclusion criteria included having no evidence of cognitive, psychological or neurological conditions. In terms of cognition, to exclude the presence of cognitive impairments, the participants were required to attain the cut-off scores on the Mini-Mental State Exam (MMSE; Folstein et al., 1975; Brucki et al., 2003). In addition, they were also required not to exceed a score of 2 points on the Questionnaire of Cognitive Change (QMC8; Damin and Brucki, 2011) and a score of 7 points on the Functional Assessment of Communication Skills for Adults (ASHA-FACS; Carvalho and Mansur, 2008). Neurological and psychological status was assessed by a psychologist and neurologists and quantified using the Geriatric Depression Scale-15 (Sheikh and Yesavage, 1986; Almeida and Almeida, 1999). In terms of hearing evaluations, the participants underwent audiological assessments, including pure-tone threshold audiometry and a speech recognition threshold (SRT) test. Both tests were administered in a sound-proof booth using a GSI Audiometer. They were required to demonstrate that they had no hearing deficits other than mild presbycusis (≤40 dB HL for octave frequencies from 250 to 8000 Hz) and similar hearing levels in both ears (e.g., no more than a 10 dB difference between the hearing thresholds of the two ears at each frequency tested).

The characteristics of the participants are described in Table 1. Variables such as age, speech recognition thresholds (SRTs) and income level were controlled. This last variable was indexed based on the family income questionnaire; the higher the score, the lower the participant's income level.

**Table 1:** **Group characteristics**.

**Variables**	**Mean ± SD**	**Minimum / Maximum**
**GENDER (n)**		
Female (#)	123	
Male (#)	54	
Age	63 ± 8.2	44/87
Years of formal schooling	9.7 ± 5.3	0/24
Income index	3.4 ± 1.1	2.0/9.0
**AUDIOLOGICAL EVALUATION**		
SRT (dB HL)		
RE	19.7 ± 9.4	5/40
LE	20.5 ± 11.1	5/35
**COGNITIVE SCREENING**		
MMSE	27.9 ± 1.7	25/30
QMC	0.3 ± 0.6	0/2
ASHA-FACS	6.9 ± 0.09	6.3/7

SRT, speech recognition threshold; RE, right ear; LE, left ear; MMSE, Mini-Mental State Exam; QMC, Questionnaire of Cognitive Change; ASHA-FACS, Functional Assessment of Communication Skills for Adults.

### Procedures and measures

After the subjects signed the written consent form, they underwent the auditory processing tests (dichotic digit, frequency pattern, and speech-in-noise tests). A working memory test was also completed (digit span—backward recall).

#### Auditory processing tests

All auditory processing tests were administered in a sound-proof booth using a GSI 61 Audiometer, Sony Compact Disc Player, and headphones. The stimuli, which were recorded on a compact disc, were played on the CD player connected to the audiometer. This audiometer controlled the stimulus intensity at a fixed level of 50 dB SL.

##### Dichotic digit test (Pereira and Schochat, 1997)

This central auditory test assessed binaural integration skills, which represent the ability of the individual to process different stimuli that are presented to each ear at the same time. This test was composed of naturally spoken dissyllabic digits with similar syllable lengths; specifically, 4, 5, 7, 8, and 9 were used. The digits were spoken in Portuguese by a male speaker. The test included 20 trials. Each trial consisted of 2 pairs of digits (each pair presented different digits to each ear simultaneously). The individual was instructed to listen carefully and repeat the 2 pairs of digits at the end of each trial. In total, the test included 40 pairs of digits (80 digits per ear). Performance was measured according to the percentage of correctly repeated digits in each ear, irrespective of the order.

##### Speech-in-noise test (Pereira and Schochat, 1997)

This central auditory test assessed speech perception in noise. The test was composed of 25 monosyllabic words that were spoken in Portuguese by a male speaker and presented to each ear at a fixed signal-to-noise ratio of +20 dB (words were presented at 50 dB SL and noise at 30 dB SL). White noise was used for the background noise. The same list of words was presented to each ear. The right ear was the first to be tested, followed by the left ear. The individual was instructed to listen carefully to each word and repeat it. Performance was measured according to the percentage of correctly repeated words in each ear.

##### Frequency pattern test (Musiek and Pinheiro, 1987)

This central auditory test assessed skills related to auditory temporal processing, which is the ability to process nonverbal auditory signals and to recognize the orders or patterns of the presentations of the stimuli. The test consisted of 20 trials with intervals of approximately 6 s between each trial pair. Each trial included three stimuli for 150 msec and an interstimulus interval of 200 ms. The low stimulus (L) was 880 Hz, and the high stimulus (H) was 1122 Hz. The individual was instructed to listen carefully to all three stimuli and to respond by naming them in the order in which they were presented (e.g., “low, low, high;” “high, low, low;” etc.). After the study, we calculated the percentage of correct answers. This test was administered diotically.

#### Working memory test

##### Digit span (backward recall; Wechsler, 1987)

This test was taken from the WAIS (Wechsler Adult Intelligence Scale) test to investigate the extent at which auditory processing and cognitive performance were associated. In this working memory test, participants were instructed to verbally repeat a sequence of numbers, also presented verbally, in the reverse order. The number of digits in the sequence was gradually increased until the participant could not repeat them correctly. The digit span performance was taken as the longest list of numbers repeated accurately.

#### Statistical analyses

Pearson's correlation and stepwise multiple regression were calculated to determine the strength of the association between years of schooling, auditory processing and working memory tests. One-way analysis of variance (ANOVA) was used to determine the gender effect.

## Results

The strength of the association between years of schooling and performance on each of the auditory and working memory tests was investigated using correlation and regression analyses. A correlation between auditory processing and working memory tests was assessed to determine the extent of the relationship between sensory and cognitive skills. Possible confounding factors such as age, income and hearing (SRTs) were controlled. One-way ANOVA revealed that the men performed significantly better than the women on the frequency pattern test [*F*_(1, 164)_ = 5.89, *p* = 0.01].

### Relationship between years of schooling and performance on the auditory processing and working memory tests

**Figure 1:**
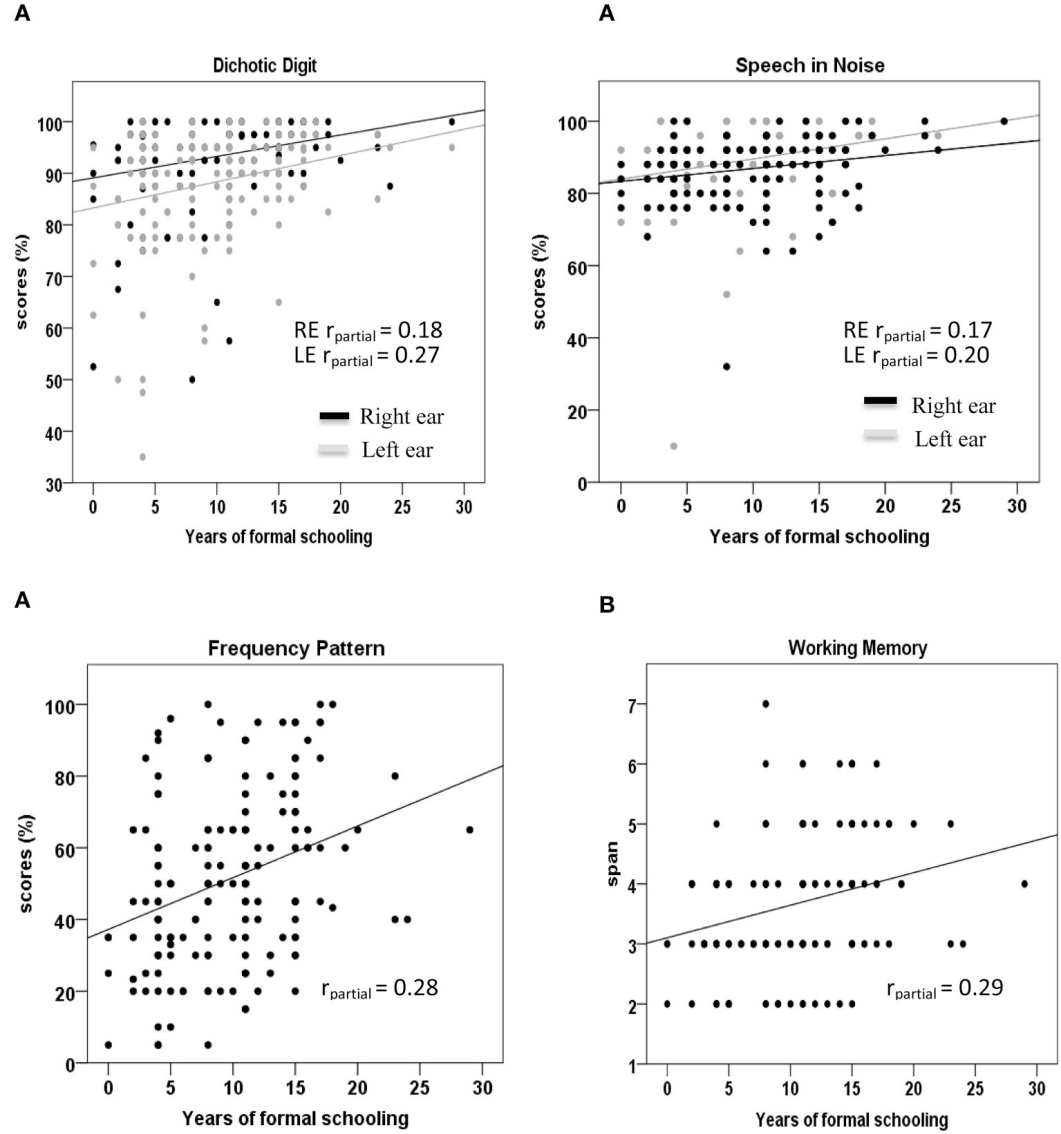
**Scatter plots of years of schooling vs. (A) auditory processing tests and (B) working memory test**. Significant (*p* < 0.05) correlation coefficients (r) for all participants with age, income, and hearing partialled out, are demonstrated in each box.

The association between years of schooling and performance on each test was assessed for the whole group using partial correlation, partialling out the effects of age, income, and hearing (SRTs). The significance of correlation coefficients was set at *p* < 0.05. The partial correlations showed a significant but weak association between years of schooling and dichotic digit [right ear: *r*_*partial*_ = 0.18, *p* = 0.03; left ear: *r*_*partial*_ = 0.27, *p* = 0.002], speech-in-noise [right ear: *r*_*partial*_ = 0.17, *p* = 0.05; left ear: *r*_*partial*_ = 0.20, *p* = 0.02] and frequency pattern test performance [*r*_*partial*_ = 0.28, *p* = 0.001]. Similar to auditory processing measures, there was a significant but weak association between years of schooling and digit span [*r*_*partial*_ = 0.29, *p* = 0.001]. The scatter plots in Figure 1 show these significant correlations as well the coefficients for the whole group when age, income and hearing (SRTs) were partialled out.

### Relationship between working memory and auditory processing tests

The association between working memory and performance on each auditory processing test was also assessed for the whole group using partial correlation, partialling out the effect of age, income and hearing (SRTs). The significance of correlation coefficients was set at *p* < 0.05. The partial correlations showed a significant but weak to moderate association between performance on the digit span and frequency pattern tests [*r*_*partial*_ = 0.40, *p* < 0.001], and a weak association between performance on the digit span and dichotic digit test [left ear/*r*_*partial*_ = 0.29, *p* = 0.001]. No significant correlations were observed between performance on the dichotic digit test in the right ear [*r*_*partial*_ = 0.15, *p* = 0.07] and performance on the speech-in-noise test for both ears [right ear/*r*_*partial*_ = −0.03, *p* = 0.7, left ear/*r*_*partial*_ = 0.05, *p* = 0.5].

**Figure 2:**
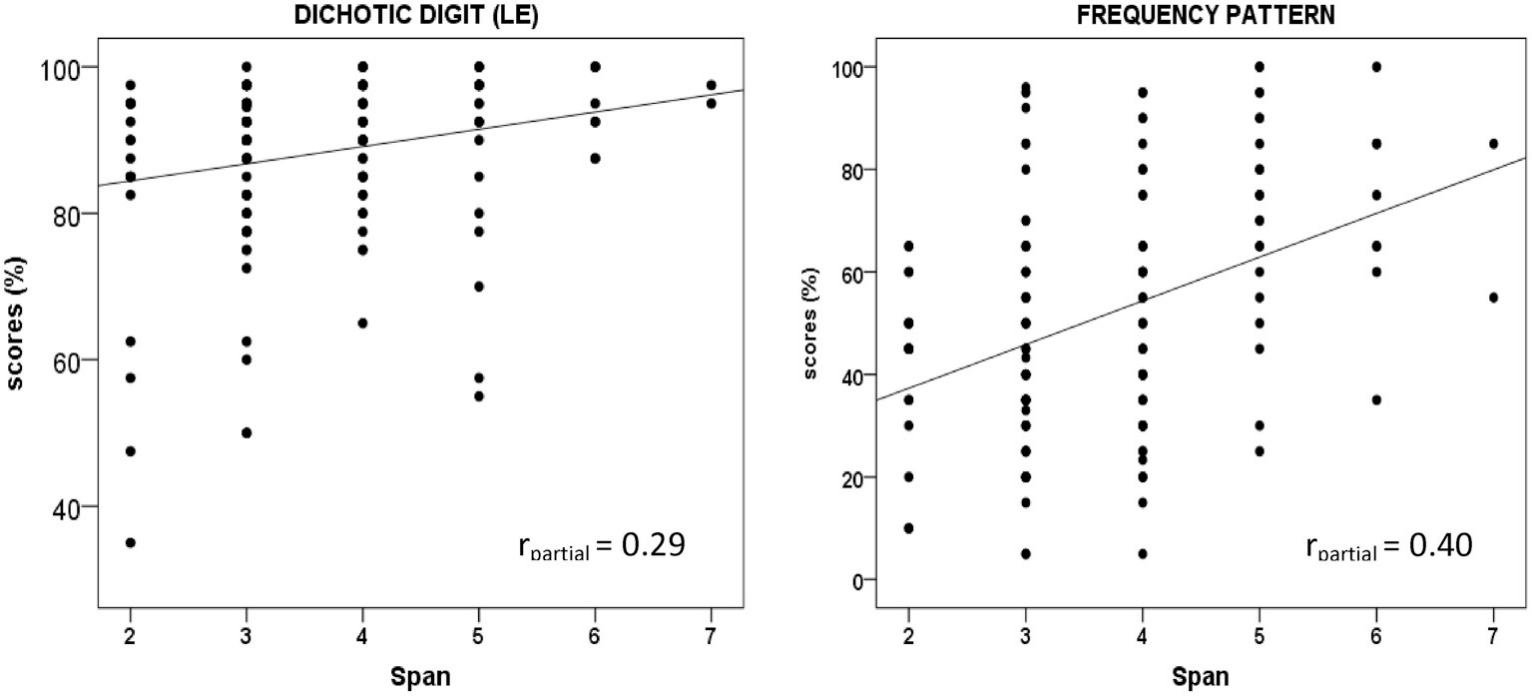
**Scatter plots of digit span vs. auditory processing tests (dichotic digit/LE and frequency pattern test)**. Significant (*p* < 0.05) correlation coefficients (r) for all participants with age, income, and hearing partialled out, are demonstrated in each box.

The scatter plots in Figure 2 show the significant correlations between performance on the auditory processing tests (frequency pattern and dichotic digit) and the working memory test. The figure also shows the significant coefficients for the whole group when age, income and hearing (SRTs) were partialled out.

### Multiple regression analysis

Multiple regression analyses (stepwise method) were performed to investigate the relative contribution of factors on the variance of the auditory processing tests.

Years of schooling, age, income, hearing scores, and working memory were considered predictor variables. Additionally, to investigate whether the auditory processing skills were related to each other, we also included each one as a predictor variable. Levels of *F* to enter and *F* to remove were set to correspond to p levels of 0.005 and 0.01, respectively, to adjust for familywise alpha error rates associated with multiple significance tests.

For the speech-in-noise test (LE), the final model, which explained the highest percentage (33%) of the variance was based on the following variables entered in the following order: speech–in-noise (RE), hearing (RE), years of schooling, frequency pattern, and dichotic digit (LE) [*F*_(5, 160)_ = 15.3, *p* < 0.001]. The standard regression coefficient indicated a positive correlation for all variables except for hearing, indicating that the higher the SRT, the worse the speech-in-noise performance. The coefficients were 0.26 (*p* < 0.001) for years of schooling, −0.23 (*p* = 0.001) for hearing (SRT on the right ear), 0.21 (*p* = 0.004) for frequency pattern, 0.14 (*p* = 0.04) for dichotic digit (LE), and 0.23 (*p* = 0.003) for speech in noise for the right ear.

For the frequency pattern test, the final model included the following variables in the following order: working memory, years of schooling, speech in noise (LE), dichotic digit (RE), and speech in noise (RE). The model explained 31% of the variance [*F*_(5, 160)_ = 14.3, *p* < 0.001] and all correlations were positive. The standard regression coefficient was 0.32 (*p* < 0.001) for years of schooling, 0.28 (*p* < 0.001) for working memory, 0.20 (*p* = 0.007) for dichotic digit (RE), 0.19 (*p* = 0.012) for speech in noise (RE), and 0.20 (*p* = 0.007) for speech in noise (LE).

For the dichotic digit (both ears) and speech-in-noise (RE) tests, years of schooling did not significantly contribute to the variance in performance.

Table 2 shows the values that were significantly related to the final regression model of each one of the auditory processing tests.

**Table 2:** **Final regression model**.

**Dependent variables**	**Predictors**	***b***	***SE b***	**β**	***R^2^* change**
Speech in noise (LE)	Speech in noise (RE)	0.26	0.08	0.23^**^	0.19
	Hearing (RE)	−0.27	0.08	−0.23^**^	0.05
	Years of schooling	0.48	0.13	0.26^***^	0,04
	Frequency pattern	−0.08	0.03	−0.21^**^	0.02
	Dichotic digit (LE)	0.12	0.06	0.14^*^	0.01
Frequency pattern	Working memory	6.20	1.54	0.28^***^	0.15
	Years of schooling	1.44	0.32	0.32^***^	0.06
	Speech in noise (LE)	−0.48	0.18	−0.20^*^	0.05
	Dichotic digit (RE)	0.56	0.20	0.20^**^	0.02
	Speech in noise (RE)	−0.56	0.22	−0.19^*^	0.02

* p < 0.05,

** p < 0.01,

*** p < 0.001

The R^2^ change reveals the proportion of the variance accounted for as predictors are included to the model.

## Discussion

The purpose of the present study was to assess the auditory processing skills of adults and elderly people with different levels of formal education. Performance on all the auditory processing tests was correlated with years of schooling. Moreover, regression analyses also confirmed the relative contribution of educational level to the performance variance for the frequency pattern and speech-in-noise tests, indicating, to some extent, that education and auditory processing skills are correlated. Performance on the working memory, frequency pattern and dichotic digit tests were also correlated.

The first important result is that, as expected, working memory skill was associated with years of schooling. This result corroborates previous findings (Blum and Jarvik, 1974; Gurland et al., 1983; Farmer et al., 1995; Brucki et al., 2003; Souza-Talarico et al., 2007) and suggests that higher education is associated with improved cognitive function. Years of schooling was also associated with performance on all of the auditory processing tests, suggesting that a higher educational level also correlated with improved auditory processing skills, as demonstrated, to some extent, by the regression results. Moreover, the significant correlation between auditory processing and working memory performance suggests that both functions partially depend on the same neural networks, which can lead to sensory-cognitive interactions across auditory and cognitive tasks and functions. Previous research has also demonstrated this sensory-cognitive interaction through the correlation between working memory tests and auditory processing skills, such as speech-in-noise perception (Pichora-Fuller et al., 1995; Akeroyd, 2008; Füllgrabe et al., 2015), pitch pattern frequency (Mukari et al., 2010) and dichotic listening tests (Hällgren et al., 2001), as observed in the present study.

Considering the correlation between performance on most of the tests and years of schooling, two main hypotheses might be considered. The first hypothesis is related to the biological explanation that the greater performance of individuals with a higher educational level is due to environmental enrichment, which might lead to an increase in the number of synapses and in vascularization and, consequently, to changes in the structure of the brain early in life (Speisman et al., 2013). This “cognitive reserve theory” is primarily associated with cognitive aspects; however, based on the results of studies of sensory-cognitive interaction and the present results, it is worth better investigating the extent to which this idea might be generalized to auditory processing skills. The second hypothesis that could explain the present results is related to the negative impact of poor auditory processing skills on learning. Several studies have demonstrated the importance of auditory processing on literacy skills by showing it is a prerequisite for learning to read and write successfully (Tallal, 1980; Murphy and Schochat, 2011; Hornickel et al., 2012; Rogowsky et al., 2013; Murphy et al., 2015). Thus, individuals with difficulties involving auditory processing could struggle academically, resulting in the completion of less formal schooling than individuals who perform better on auditory processing tests.

Although it might be assumed that “years of schooling” was generally an important factor for auditory processing performance, for the dichotic digit test, in particular, years of schooling was not one of the best predictors of performance. This result, as well as the presence of different predictors for each one of the tests applied, suggests that the dichotic digit test, speech-in-noise test and frequency pattern tests assess different skills to a certain extent.

The frequency pattern test, for instance, is likely the most cognitively demanding test, resulting in the strongest correlation with working memory test and in the identification of working memory as one of the best predictors of performance. These results corroborate previous studies that also demonstrated the influence of working memory on auditory temporal processing performance (Mukari et al., 2010; Broadway and Engle, 2011; Füllgrabe et al., 2015). This cognitive influence might underlie the association between performance on this test and the level of education. Curiously, performance on this test was also affected by gender. This result was not expected and requires further investigation in future studies. For the speech-in-noise test, in addition to years of schooling, hearing was one of the best predictors of performance, corroborating the sensory peripheral hypothesis regarding the auditory processing difficulties of the elderly (Humes et al., 2012). According to this hypothesis, auditory difficulties, such as those related to understanding speech in background noise, are predominantly the consequence of the loss of audibility associated with age-related hearing loss, which leads to a causal interaction between central and peripheral auditory deficits. “Years of schooling” was one of the best predictors for only the left ear in the speech-in-noise test. Perhaps, this result was not associated with the ear *per se* but rather with the order in which the stimuli were presented. This is because, in tests such as the speech-in-noise test, the same list of words is presented twice, once for each ear. Consequently, it is expected that the second ear will perform better than the first ear given the individual's previous knowledge of the linguistic material, which might be used as a cognitive strategy (Pereira and Schochat, 1997; Oliveira et al., 2013). Thus, in the present study, the influence of years of schooling for only the second ear (the left ear) might indicate that the higher the level of education, the better the use of this cognitive strategy. “Years of schooling” was not one of the best predictors of dichotic digit performance even though they were correlated. This result may be related to the fact that this test is not sufficiently cognitively-demanding not only in terms of working memory but also in terms of linguistic aspects.

Performance on individual auditory processing tests was correlated with performance on the other auditory processing tests, suggesting that, to some extent, the same underlying components are being assessed. These shared components likely involve auditory perceptual skills and more sensory aspects related to the biological basis of ascending auditory system function. Despite that, the results for both frequency pattern and speech-in-noise test indicate that educational level is likely related to the cognitive demand of the auditory processing tests rather the auditory sensory aspects itself. Thus, perhaps differences in working memory and linguistic demand might explain the different degrees of the contribution of education to performance on each of the tests. From a clinical perspective, the present findings demonstrate that individual's educational level must be taken into consideration for auditory processing tests not only at the time of the diagnosis of auditory processing disorder but also in the normalization of the auditory processing tests.

This study has several limitations. First, it is a cross-sectional study; thus, longitudinal studies are required to better investigate both of the hypotheses highlighted previously. Another limitation of the present study is the lack of information regarding the music skills of the participants, which can be considered as a confounding factor. Further studies should consider this factor as an exclusion criterion on the selection of the participants.

## Conclusions

The present findings suggest that educational level is partially associated with performance on auditory processing tests. However, this association is likely due to the cognitive demand of the auditory processing tests rather than auditory sensory aspects itself. Further studies should investigate the influence of education using auditory electrophysiological tasks that are more complex and determine the extent to which educational levels might slow auditory processing declines using longitudinal studies.

## Author contributions

Designed the experiments: CM, ES. Performed the experiments: CR, MS. Analyzed the data: CM. Wrote the paper: CM, CR, MS, LM, ES.

## Funding

Financial support: CNPq (Conselho Nacional de Desenvolvimento Científico e Tecnológico) Grant number: 557887/2009-9.

### Conflict of interest statement

The authors declare that the research was conducted in the absence of any commercial or financial relationships that could be construed as a potential conflict of interest.
